# Smaller Cortisol Awakening Responses Are Associated with Greater Visual Dependence in Postural Control

**DOI:** 10.3390/healthcare9060723

**Published:** 2021-06-12

**Authors:** Nina Smyth, Monica Milani, Lisa Thorn, Maria Flynn, John F. Golding, Phil Evans, Angela Clow

**Affiliations:** School of Social Sciences, Psychology, University of Westminster, 115 New Cavendish Street, London W1W 6UW, UK; mncmilani@gmail.com (M.M.); L.Thorn01@westminster.ac.uk (L.T.); flynnm@westminster.ac.uk (M.F.); goldinj@westminster.ac.uk (J.F.G.); p.d.evans@westminster.ac.uk (P.E.); clowa@westminster.ac.uk (A.C.)

**Keywords:** salivary cortisol, cortisol awakening response, hypothalamic–pituitary–adrenal (HPA) axis, postural control, visual dependency

## Abstract

There are known links between the hypothalamic–pituitary–adrenal (HPA) axis and systems responsible for regulating posture. Our aim was to explore directly, for the first time, whether an aspect of circadian HPA axis activity (the cortisol awakening response: CAR) was associated with greater visual dependency in postural control. For measurement of the CAR, electronically monitored saliva samples were collected by participants following morning awakening in their home environment. On the afternoons of the same days, postural sway was measured in the laboratory by exposing participants to static (control) and moving visual stimuli whilst standing still and upright on a force platform. Visual dependence was assessed as the increase in postural sway (path length) during exposure to the moving compared with the static condition. The 44 measurement days were derived from four days for each of eleven healthy participants (mean ± SD age: 51.18 ± 3.3 years). As expected, postural sway was greater when exposed to moving versus static cues. Mixed regression modelling showed that participants with smaller four day average CARs had greater deterioration in postural sway when presented with moving stimuli. These data are the first to document associations between the CAR and visual dependency in postural sway.

## 1. Introduction

Postural control (i.e., balance) is maintained by a complex integration of inputs from the semi-circular canals in the ear, proprioceptive (physical feedback from muscles and joints) and visual systems. There are individual differences in the degree to which these systems are used for maintaining postural control [1,2]. Visual dependency is the greater use of visual cues and it is normally distributed in the general population [3]. Individuals who are more visually dependent can be more likely to experience postural instability when exposed to complex-moving stimuli in the environment [4,5]. Visual dependency is associated with increasing age and ill health [5,6]. Visual dependency can be measured in the laboratory by presenting moving visual stimuli whilst an individual is standing stationary and upright on a force platform to measure postural sway. The extent to which visual stimuli increases postural sway compared to a static stimulus (control condition), provides a measure of visual (or field) dependency in postural control [5]. Such situationally specific increased body postural sway has been proposed to be a key concept for understanding the relationship between postural control and associated conditions [7]. The direction of causality in these relationships is not known but it has been proposed that they are mediated by a shared underlying disturbance [8,9].

There are known bi-directional anatomical links between postural control systems and the hypothalamic–pituitary–adrenal (HPA) axis [3,10]. The HPA axis is activated under stress with secretion of cortisol as its end-point [11]. Psychological stress can influence postural control [10]. Secretion of cortisol from the HPA axis cascade is regulated across the 24 h day via projections from the suprachiasmatic nucleus (SCN) to the paraventricular nucleus of the hypothalamus [12]. This pathway generates a typically healthy circadian pattern of cortisol secretion characterised by a nadir during night-time sleep, gradually rising levels prior to morning awakening and a marked cortisol awakening response (CAR) followed by a gradual diurnal decline. The CAR normally peaks within 30–45 min post-awakening. Importantly, it is a ‘dynamic’ response to awakening, which differs from total (mean) cortisol levels in the post-awakening period [13]. Healthy circadian cortisol secretion is important to synchronise ‘slave clocks’ throughout the brain and body [14] and disruption of this system may be an underlying cause of the development of psychopathology [15]. Thus, the CAR may be a particularly important circadian pacemaker for the brain and peripheral organs as it receives regulatory control from two pathways from the SCN [12]. Chronic activation of the HPA axis dysregulates the CAR [11]. The CAR is a ‘*window on the brain*’ as dysregulation of it provides early evidence of dysregulated brain function and is a key pathway linking stress and disease [16]. It can be accurately measured within the domestic setting by self-collection and electronic monitoring of saliva samples [13,17].

There are no reported studies examining the CAR in relation to postural control. Its role as a sensitive marker of circadian HPA axis activity [12] provides a rationale for investigation in relation to postural control. Although we do not suggest a simplistic causal link between the CAR and postural control, we postulate that examination of the CAR, would reveal associations between the two systems in healthy participants with no diagnosed postural disorder. Understanding these links in healthy individuals is theoretically interesting and potentially useful in terms of the development of novel intervention for strategies to maintain postural control.

Here, we sought to examine, for the first time, the relationship between visual dependence in postural control and the CAR in healthy middle-aged adults. The CAR was assessed in line with best practice guidelines [13]. It was hypothesised that participants with smaller averaged CARs would show greater average deterioration in postural sway when exposed to moving stimuli compared to a static image.

## 2. Materials and Methods

### 2.1. Participants

Healthy adults (N = 11: 6 females, 5 males; aged 51.18 ± 3.3 years) not suffering from any medical or psychiatric conditions or taking steroid prescribed medication were recruited onto this study. Participants self-reported good health and one participant was a smoker. Participants gave full written consent and this study was approved by the University of Westminster Ethics Committee (Ref: 1516-1354).

### 2.2. Design

The CAR was measured under naturalistic settings, in participants’ home environment. The CAR is known to be variable across days [13], so in order to obtain a reliable individual average, it was necessary to measure it across several days. Thus, the CAR was measured on 4 study days. Postural control was measured in the afternoon of the same day as CAR assessment. As a previous study has shown that a bigger CAR, measured across 4 days, each 1 week apart, predicted brain activity [18], a similar study design was adopted for the current study. Study days were each one-week apart to obtain measurement of CAR and postural sway on distinct days.

### 2.3. Procedure

**Figure 1:**
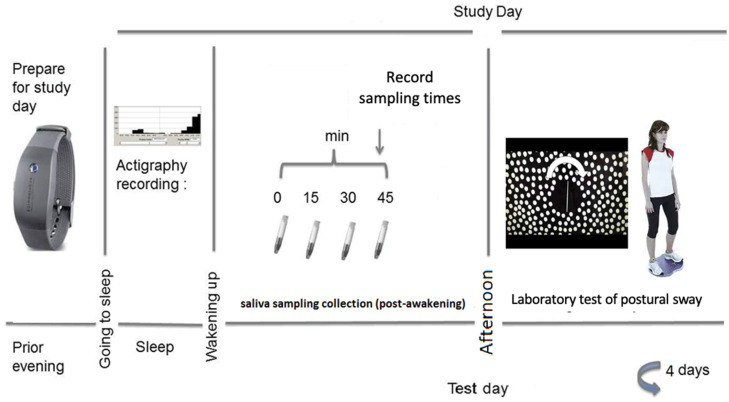
Timeline of protocol for measurement of the CAR and postural sway.

Participants had a one-to-one induction with the researcher, where the study protocol was explained, including the importance of adherence to the saliva sampling protocol. Participants were also able to practice the posture test (see laboratory testing below) and collection of a saliva sample. On four weekdays (one week apart), participants collected saliva samples at 0, 15, 30 and 45 min post-awakening (samples 1–4). Participants were instructed to awake in their usual way and to collect the first awakening sample immediately on awakening. During the saliva collection period, participants were instructed to remain nil-by-mouth (except water). Participants were asked to complete a record sheet each day to report awakening and sampling times. Participants were reminded about upcoming study days and to prepare saliva sampling materials the evening before via an automated mobile phone text message. In the afternoons (between 13:30 and 16:30), participants attended the laboratory for approximately 30–45 min for the laboratory tests of balance (timings were consistent for each participant). Saliva samples were initially stored in a domestic freezer until they were returned to the laboratory to be stored at −20 °C until assayed. See Figure 1 for an overview of the protocol for CAR assessment and postural sway.

### 2.4. Saliva Samples and Electronic Monitoring of the Saliva Sampling Protocol

Participants were provided with full-standardised written instructions and were able to contact the researcher if they had any questions about the sampling protocol. Pre-labelled salivettes (saliva sampling devices, Sarstedt Ltd., Leicester, UK) were provided in four Ziploc bags for each study day. Samples were labelled as awakening sample (sample 1), 15 min (sample 2), 30 min (sample 3) and 45 min (sample 4). Participants were provided with a record sheet to record their awakening and saliva sampling collection times. Participants were also provided with electronic devices used to monitor awakening times (wrist-worn Actiwatch, Cambridge Neurotechnology, Cambridge, UK) containing piezo-electric motion sensor recording physical activity. Awakening times were estimated using the actiwatch software that distinguishes sleep and awakening periods by reduced and increased activity, respectively. In line with recommendations [19], actigraph awakening times were scored by the human eye rather than the computer algorithm. One researcher (MB) scored all awakening times and the lead researcher (NS) verified at least 10% of these. To monitor saliva sampling collection times, the cotton swabs (for collection of saliva) were stored in an electronic bottle (medication event monitoring: MEM cap). Participants were instructed to open the bottle only at sampling times. Following saliva collection, swabs were returned to the correctly labelled salivette for storage. Both electronic devices were used to verify awakening and sample collection times.

### 2.5. Laboratory Testing: Visual Dependence in Postural Sway Assessment

Participants stood still in front of a display screen (height 180 cm, width 240 cm size), at approximately 120 cm distance. Artificial room light was standardised (to ensure consistency in presentation of the visual stimuli), with sunlight restricted with blackout blinds. Sway path length was measured on a stable force platform (Accusway AMTI Ltd., Watertown, MA, USA). Total sway path length was measured in cm and divided by the duration of trial to obtain average sway path length (cm/s). Whilst viewing the display panel, participants were instructed to stand on the force platform with feet together, and arms by their sides. The standing position on the first testing day of each participant was recorded by drawing around the feet on a paper template. This personalised template was used on each subsequent study day to ensure a consistent stance over the four study days. The display panel presented a stimulus for 60 s during which time sway path length was measured. Sway was measured during two conditions, static (i.e., no visual moving image: control) and motion (i.e., moving visual image); see Figure 1. During each trial, participants wore a pair of lightweight goggles in order to focus gaze on the stimulus and to restrict peripheral visual cues which could cause distraction or provide additional uncontrolled information. Participants completed the Rod-and-Disk task [20,21]. The task involved viewing an image filled with a collage of 220 off-white dots, each 8 mm (1.5° of visual field) in diameter, randomly distributed on a black background.

### 2.6. Cortisol Assessment and Assay

Saliva samples were thawed and centrifuged for 10–15 min at 3500 rpm. Cortisol concentrations were determined by enzyme-linked immunosorbent assays (Salimetrics LLC, State College, PA, USA) at the Psychophysiology and Stress Research Group’s laboratory. Standards, controls and all samples were assayed in duplicate and intra and inter-assay variations were both below 10%.

### 2.7. Treatment of Data and Statistical Analysis

Analyses were conducted on SPSS version 25, IBM Corp. Armonk, NY, USA). Values were moderately positively skewed for postural sway and transformed to normalise sample distributions for inferential analyses. The CAR was calculated as the mean increase (MnInc) of subsequent samples (s2–s4) from the first sample (s1) taken at awakening ([s2 + s3 + s4] / 3 − s1).

Saliva sampling times were determined electronically and calculated as the interval between awakening and collection of sample 1, with protocol timings added for samples 2–4, in line with recommendations [17]. Delays (>15 min) between awakening and collection of the awakening sample (n = 1 day) were removed from all analyses. Analyses were conducted on a total of 43 days.

Mixed regression modelling was used to investigate the effect of visual stimulus condition (static vs. motion) and MnInc on postural sway. Data were standardised as is the usual procedure for mixed regression modelling to improve model interpretability. In all models, participant identity was the subject variable and temporal order of study days and visual stimulus condition were modelled as repeated effects. Models were initially run to optimise the covariance structure for repeated measures expressed by day and sample-point order. A first-order auto-regressive structure was adopted in the final model based on tests of covariance parameters and minimising of Schwarz’s Bayesian Criterion (BIC). In model A, the effect of visual stimulus condition (static vs. motion) on postural sway was tested and in model B the modulating effect of the MnInc on postural sway was also tested. Additional analyses were conducted to control for potentially confounding individual difference variables known to influence the CAR. Subsidiary analyses were also conducted excluding delays over 3 min or when samples 2–4 deviated ±7.5 min from the 15 min timing protocol, in line with recommendations [17].

## 3. Results

**Figure 2:**
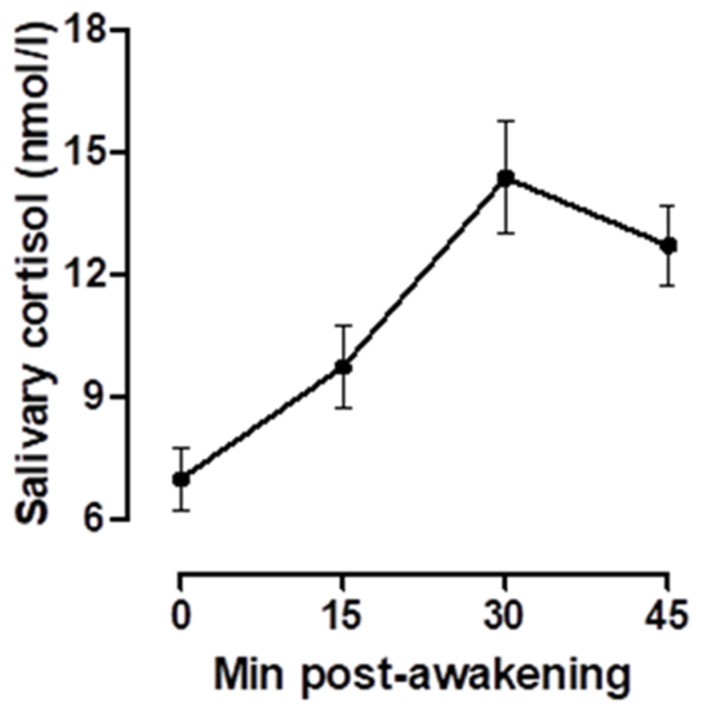
Mean (±SEM) salivary cortisol concentrations post-awakening.

Raw values for cortisol concentration ranged from 0.58 to 41.67 nmol/L and for postural sway path length between 0.65 and 5.84 cm/s. On average, across all days and participants, cortisol concentrations exhibited the typical rise from the awakening sample, peaking at the 30 min sample and increasing between 6.99 and 14.38 nmol/L. Figure 2 illustrates the average cortisol concentrations across days and participants. Across all days and participants, the mean MnInc was 5.29 (standard error mean: SEM = 0.33). Across all days and participants, verified median wake time (determined by actigraphy) was 06:01 hh:mm (interquartile range: IQR: 05:59–06:49 hh:mm). Participants were highly accurate to the saliva sampling protocol, with a median interval of 2 min (IQR = 1–3 min) between awakening and collection of the awakening sample.

**Figure 3:**
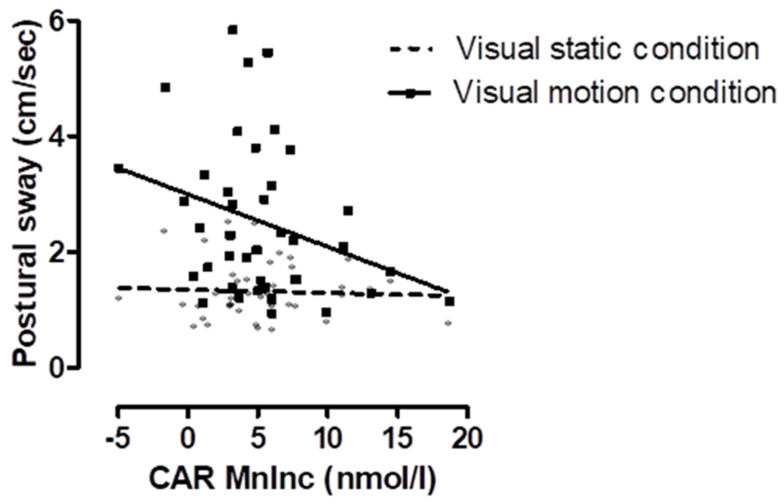
Interaction between standardised visual stimulus condition: static (grey diamond symbol) versus motion (black square symbol) and cortisol awakening response on postural sway (log cm/s).

In model A, the effect of visual stimulus on postural sway was tested (see model A in Table 1); in the motion condition (M ± SD = 2.10 ± 1.32 cm/s), in comparison to the static condition (M ± SD = 1.32 ± 0.48 cm/s), postural sway was significantly greater (F = 127.501, df = 1, 50.715, *p* < 0.001). In model B, the MnInc as a modulator of the effect of visual stimulus condition (static vs. motion) on postural sway was tested (see model B in Table 1). A significant interaction between condition and the MnInc was observed (F = 12.787, df = 1, 53.909, *p* < 0.001), indicating that sway was greater in the motion condition when the MnInc was lower (see Figure 3). The effect size was R^2^ = 0.191 and power estimate of 0.943.

A series of additional analyses were undertaken to explore whether simultaneous entry of potentially relevant covariates (awakening-time, sampling inaccuracy, sex, medication, and smoking status) into the models might significantly change the observed results. Findings were robust to these tests of extraneous covariate influence. Additionally, analyses were repeated only on days in which sampling relative to awakening was deemed short (i.e., within 3 min of awakening) and when sampling between sample 2 and 4 was ±7.5 min from the 15 min protocol timing (n = 6) were removed, and the main results were unchanged.

## 4. Discussion

Postural stability is maintained by a complex integration of inputs from the semi-circular canals in the ear, proprioceptive and visual systems. Disorientating visual inputs, as used in this study, lead to increased postural sway because one regulatory input (i.e., the visual system) was challenged. Some people are better at compensating with the alternative inputs than others. In line with our hypothesis, we show that the CAR, a sensitive marker of circadian HPA axis activity, is associated with visual dependency in postural sway (i.e., greater postural sway when visual inputs are challenged) in healthy middle-aged individuals. Individuals with smaller average CARs showed greater visual dependency in postural sway. Those with greater CARs, representing a healthy, functional response, did not exhibit increased postural sway under visual challenge. Prior work has only shown how HPA axis activation in terms of overall cortisol secretion [22,23] and acute stress cortisol responding to a stressful situation [24] were related with visual dependency in postural sway. The association found in the current study does not indicate a simplistic direct causal pathway between the CAR and postural control. Rather, it provides evidence of an overlap between the complex regulatory systems of the HPA axis and postural control. The findings are theoretically interesting and potentially of use in terms of novel intervention development.

Bi-directional anatomical links between psychological stress, HPA axis and the postural control system [3,10] make the observed associations between an aspect of circadian cortisol secretion and visual dependence plausible, although the direction of causality is not clear. Although the CAR and visual dependency have not previously been measured simultaneously in the same individuals an attenuated CAR has also been associated with a range of other conditions linked to greater visual dependency in postural sway. For example, anxiety and type 2 diabetes manifest with greater visual dependence in postural control [5,6] and attenuated CARs [25,26]. These associations provided the rationale for the current investigation and may indicate a role for the CAR in regulation of visual dependency in postural sway, which may be important in the range of conditions where this is a risk factor for poor prognostic outcomes.

We found a highly significant effect and provide effect size and power estimate. Although effect size based on smaller samples are less likely to match the true effect and observed power may thus be inflated for this kind of study, it is unlikely that the significant finding in this case represents a type I error for a variety of reasons. Firstly, the current findings are in line with previously reported findings from our lab showing a link between postural sway and HPA axis activity [24]. Secondly, power to test a hypothesis is only as good as reliability and validity of key measures, and this study was designed such that both postural sway and crucially the CAR were measured in line with best practice guidelines and repeated measurement on four distinct study days. Thirdly, the mixed (hierarchical) modelling approach used in the current study involves conducting analyses on participant-days, where analyses are conducted on the product of days and persons. These crucial repeated assessments, in terms of addressing temporal stability of measures, increase power to reveal the predicted between-subject effects.

This study was judiciously designed and carefully executed; healthy individuals were studied on four separate days, at least one week apart, to increase the reliability of both CAR and postural sway measurement. The CAR expert consensus guidelines [13] were followed; notably measuring the CAR on several days, careful electronic monitoring of awakening and sampling times and using mixed regression modelling which is robust to saliva sampling delay if assessed in this way. Such provision of CAR measurement is required to ensure accurate measurement in order to understand its role in disease [13,17]. Given the sex differences in postural control and the CAR [13,27], it would be worthwhile to examine whether there are sex differences in this relationship and in a larger sample. Moreover, examining relationships between visual dependency and other measures of circadian cortisol secretion, such as the diurnal decline, are important as this is also implicated in stress [11]. It would also be very interesting to examine these relationships in older age and in clinical populations (e.g., with vestibular dysfunction and mild cognitive impairment). Understanding better the role of cortisol circadian patterns in visual dependency in postural sway could inform intervention development for improving and maintaining postural control.

## 5. Conclusions

In conclusion, for the first time, these data indicated an association between the CAR, an aspect of the circadian pattern of HPA axis activity, and increased postural sway during visual motion (i.e., visual or field dependency). This novel finding justifies research in a larger sample to explore and confirm results found in this study. As visual dependence in postural control and attenuated circadian patterns of cortisol secretion share associations with increasing age and poor prognostic outcomes for a range of health outcomes, further investigation of the relationship between CAR and postural control in older adults and those with clinical conditions (e.g., anxiety and type 2 diabetes) is warranted.

## Figures and Tables

**Table 1:** Modelling effect visual stimulus condition (static versus motion), and cortisol awakening response on postural sway.

	Model A		Model B	
	Co-Efficient	SE	Co-Efficient	SE
Intercept	0.068	0.161	0.095	0.164
Visual stimulus condition (static vs. motion)	0.598 *	0.053	0.599 *	0.048
MnInc			−0.130	0.114
Visual stimulus condition * MnInc			−0.204 *	0.057

* <0.001.

## Data Availability

Data will be made available on request.
